# Reply: Metronomic chemotherapy with cyclophosphamide and dexamethasone in patients with metastatic carcinoma of the prostate

**DOI:** 10.1038/bjc.2012.79

**Published:** 2012-03-13

**Authors:** O A Khan, A D Blann, M J Payne, M R Middleton, A S Protheroe, D C Talbot, M Taylor, C Han, M Patil, A L Harris

**Affiliations:** 1 Department of Medical Oncology, University of Oxford, Churchill Hospital, Oxford OX3 7LJ, UK; 2 University Department of Medicine, City Hospital, Birmingham B18 7QH, UK

**Sir,**

We are pleased to see the interest of Dickinson *et al* (2012) in our paper (Khan *et al*, 2011), and note their results with prostate cancer. However, we were cautionary in terms of how to interpret our data and strongly suggest the need for appropriate randomised studies.

In their retrospective audit, Dickinson found that their patients had been more heavily pre-treated than reported in previous prostate cancer studies, and indeed, using the recommended criteria for partial response, the rate was 14%. This is a low response rate, especially in the context of new treatments that are available for prostate cancer.

It is not stated whether the patients who showed symptomatic benefit were those who showed the maximum reduction in PSA. The authors say that the steroids may not have had much influence, so it is difficult to judge why they were continued in the presence of previous progression.

There were no data on imaging blood vessel perfusion or vascular markers to show that the effects of metronomic therapy described in their study were related to anti-angiogenic effects.

We would agree that the response to metronomic therapy is likely to be strongly influenced by the origin of the primary tumour. For example, this type of regimen shows activity in other studies with breast cancer. The key point is that there is no generic effect on blood vessels of all tumour types, but a very specific effect on certain types of cancer that are known to respond well to cyclophosphamide.
